# Assessing bias in administrative database studies of RotaTeq vaccine completion due to exclusion of subjects with incomplete follow-up

**DOI:** 10.1186/s12982-015-0027-6

**Published:** 2015-04-18

**Authors:** Stephan Lanes, Scott C Quinlan, T Christopher Mast, Sander Greenland, Crystal N Holick

**Affiliations:** HealthCore, Inc., 800 Delaware Avenue, Fifth Floor, Wilmington, DE 19801 USA; Department of Epidemiology, Merck Research Laboratories, 351 N. Sumneytown Pike, North Wales, PA 19454 USA; Department of Epidemiology, UCLA School of Public Health, Los Angeles, CA 90095 USA

**Keywords:** Rotavirus vaccination, Completion rates, Bias, Follow-up

## Abstract

**Background:**

RotaTeq® pentavalent human rotavirus vaccine (RV5) is effective against rotavirus illness and rotavirus-related hospitalizations and death. Effectiveness depends on adherence to the dosing schedule, which includes 3 doses at ages 2, 4 and 6 months. Two studies have used automated claims databases to estimate the proportion of vaccinated infants who complete the dosing schedule, but excluded from analysis vaccinated infants who were not enrolled in the database for a sufficient period to observe all 3 doses. Restricting study populations based on duration of follow-up can introduce bias if a large number of subjects are excluded due to insufficient follow-up, and if their outcomes differ from subjects who are included. To address the possibility that exclusions may have been extensive and led to biased estimates of completion rates, we conducted a claims database analysis in the HealthCore Integrated Research Database^SM^ to evaluate the proportion of rotavirus vaccinated infants who completed the 3 dose series of RV5. We evaluated potential error introduced by restricting analyses to infants with complete follow-up by estimating completion rates among infants with complete follow-up, and using Kaplan-Meier analyses to estimate completion rates including infants with incomplete follow-up.

**Results:**

The inclusion criterion requiring continuous enrollment for the first year of life resulted in only 108,533 (40%) of 233,143 vaccinated infants from 2006–2012 being included in the analysis. After relaxing inclusion criteria, we were able to include 86% of vaccinated infants. The estimated completion rate among infants with continuous enrollment from birth through the first year of life was 78.1% (95% confidence limits [CLs] 77.8%, 78.3%), and among the expanded population the estimated completion rate was 77.4% (95% CLs 77.2%, 77.6%).

**Conclusions:**

These results indicate that most infants were not followed in the database through the first year of life, but the impact of excluding infants with incomplete follow-up was negligible when assessing RV5 completion rates for this commercially insured population. Nonetheless, to increase the size of study populations and reduce the potential for bias, it is preferable to include subjects with incomplete follow-up in automated database analyses, and adopt more robust approaches to defining and analyzing study populations that account for missing data.

## Background

Rotavirus is a common cause of severe gastroenteritis among infants. There are two live rotavirus oral vaccines marketed in the US, RV5 (RotaTeq® pentavalent human rotavirus vaccine, Merck & Co., Inc.) approved in 2006, and RV1 (Rotarix® monovalent human rotavirus vaccine, GlaxoSmithKline Biologicals) approved in 2008 [1]. Each vaccine is supported by the Advisory Committee on Immunization Practices (ACIP) and the vaccines are effective against rotavirus illness and rotavirus-related hospitalizations and death [1]. In a recent study, 89% of infants receiving rotavirus vaccination received RV5 [2]. The effectiveness of RV5 depends on adherence to the dosing schedule, which includes 3 doses at ages 2, 4 and 6 months of age [1]. The recommended maximum age for the first RV5 dose is 14 weeks, and the third RV5 dose should be administered by 8 months of age [1].

Two automated database studies used commercial insurance claims and estimated the proportion of rotavirus vaccinated infants completing all 3 doses of RV5 as 79% and 83% [2,3]. Because vaccination is associated with specific procedure codes and payments, claims databases should have excellent completeness in recording vaccinations for members who receive them during periods of enrollment. Claims databases represent dynamic populations, however, in which infants enter and exit the database when they obtain or terminate health insurance coverage (e.g., because their parents acquire or lose insurance coverage or change insurance carriers). Therefore, if an infant enters a database after receiving an initial vaccination, there will be no record of the first vaccination in the database. Similarly, after insurance coverage is terminated, the database will contain no evidence of any subsequent vaccinations. To address missing data due to incomplete follow-up, previous database studies restricted analyses to infants with sufficient duration of database enrollment to observe all 3 rotavirus vaccine doses if they were administered. Krishnarajah et al. [2] included infants continuously enrolled from 1 month of age through 9 months of age, and Panozzo et al. [3] included infants who were continuously enrolled from birth through 11 months of age.

Restricting study populations based on duration of follow-up can introduce bias if a large number of subjects are excluded due to insufficient follow-up, and if their outcomes differ from subjects who are included [4,5]. Neither Krishnarajah et al. [2] nor Panozzo et al. [3] provided sufficient data to estimate the percentage of infants excluded due to incomplete follow-up. To the extent that these US commercial insurance claims databases have similar durations of enrollment, our results (below) indicate that this percentage could have been very large in both studies. To address concerns that extensive exclusions may have led to biased estimates of completion rates, we conducted a claims database analysis to evaluate the proportion of rotavirus vaccinated infants who complete the 3 dose series of RV5 (completion rates), and to evaluate potential error introduced by restricting analyses to infants with complete follow-up. Specifically, we examined (a) the proportion of infants that might be excluded due to insufficient follow-up, and (b) the potential impact on RV5 completion rates of infants who are excluded.

## Methods

We conducted a retrospective study using the HealthCore Integrated Research Database^SM^ (HIRD). The HIRD is an automated database comprising medical and pharmacy insurance claims data from approximately 38 million health plan members across the United States. Member enrollment, medical care (professional and facility claims), outpatient prescription drug use, outpatient laboratory test result data, and health care utilization are tracked longitudinally for health plan members in the database since January 2006.

We identified all infants who received at least 1 dose of RV5 during the first year of life between February 1, 2006 and November 30, 2012. For the initial cohort extraction, we did not require any continuous period of enrollment or that first enrollment occur by any specific age; instead, we included any infant who had at least 1 dose of RV5 between birth date and first birthday to identify as completely as possible all vaccinated infants. Exposure to RV5 was identified using medical claims with Current Procedural Terminology (CPT) code 90680. For all infants identified, information was collected on dates of all RV5 vaccinations, geographic region of residence, and insurance coverage information.

To evaluate completion, we first identified infants with complete follow-up (complete case analysis). Among the cohort of infants having received at least 1 dose of RV5 in the first year of life, we identified infants who were continuously enrolled in the health plan from birth through their first birthday.

To include infants with incomplete follow-up, we sought to identify infants for whom the first dose of RV5 recorded in the database was in fact the first dose of RV5. We examined RV5 doses administered by infant age to examine age ranges during which different doses occurred. To exclude as few infants as possible, we determined the latest age for which we could be confident that no vaccination would have been given earlier, so that the next vaccination received would be the first dose. We then followed these infants for RV5 vaccination as long as they remained in the database.

Among infants with complete follow-up through the first year of life, we computed completion rates as the proportion of vaccinated infants receiving all 3 doses (Cohort A). Among infants who terminated health plan enrollment during the first year of life, and for whom we could determine the first dose, we computed completion rates using Kaplan-Meier analyses with right censoring upon the earliest of receipt of the third dose, termination of database enrollment, or end of the follow-up period (Cohort B). To evaluate change over time in completion rates, we stratified results according to calendar periods 2006–08 and 2009–12. The calendar period stratum was assigned according to the date the of the first RV5 dose. Confidence limits (CLs) were computed using the Wald method for binomial data [6].

## Results

**Figure 1:**
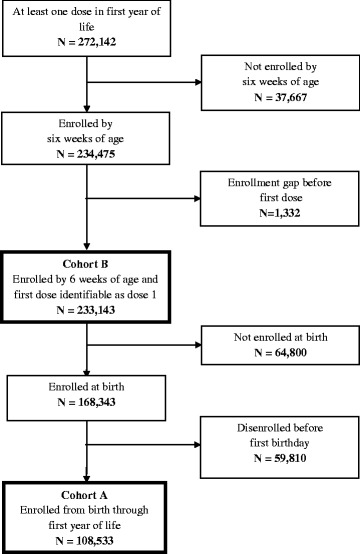
RotaTeq® (RV5) cohort formation in the HealthCore Integrated Research Database^SM^ (HIRD).

**Figure 2:**
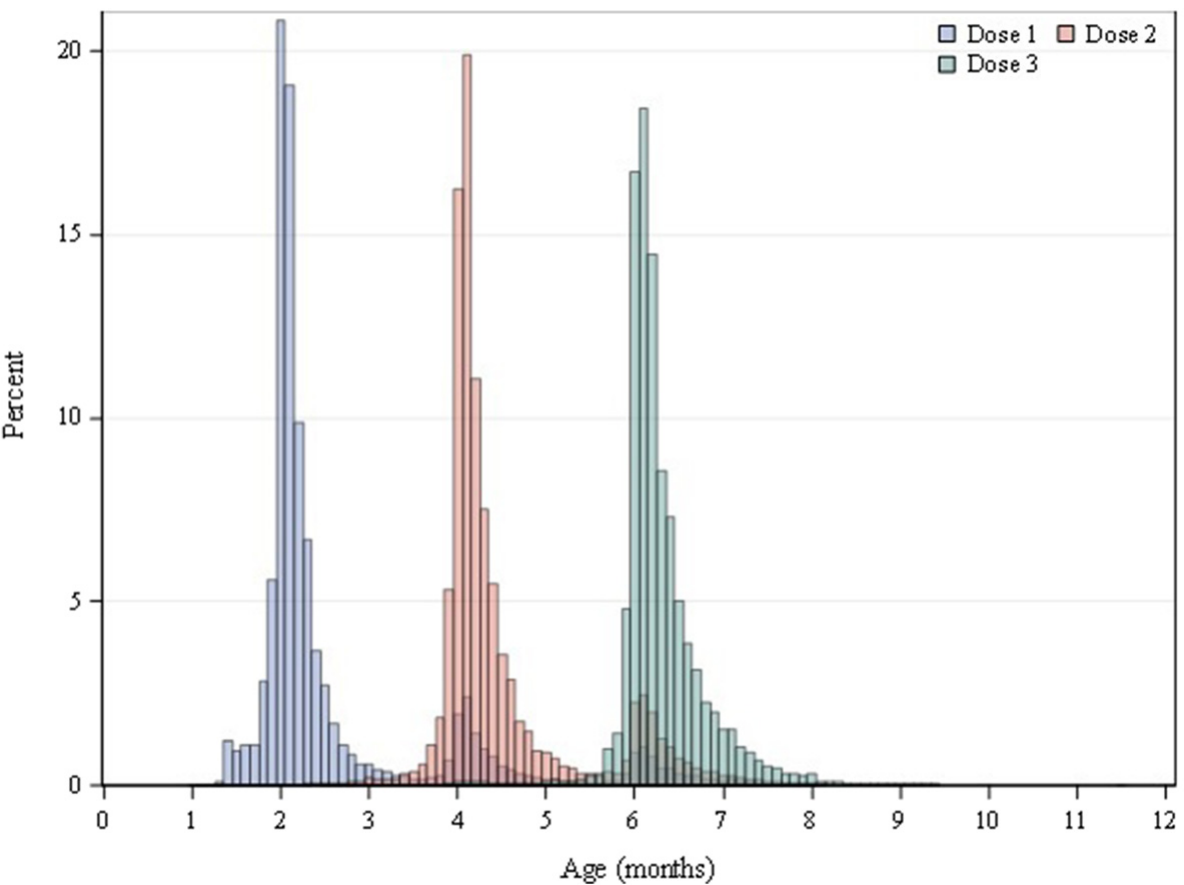
Distribution of RotaTeq® doses by age in the HealthCore Integrated Research Database^SM^ (HIRD), 2006–2012. The distribution of RotaTeq® doses by age in the HIRD among infants who received at least one dose of RotaTeq® in the first year of life, 2006–2012.

We identified 272,142 infants in the database having at least 1 dose of RV5 in the first year of life. Among these infants, we identified 108,533 (39.9%) infants who were enrolled continuously from birth through the first year of life (Figure 1, Cohort A). Cohort A was followed for 108,553 person-years. To increase the size of the study population, we examined the distribution of first doses by age to determine age cutoffs prior to which very few infants receive any doses of RV5 (Figure 2). In the overall cohort, we observed that the first dose occurred prior to 4 weeks of age for 0.02% and prior to 6 weeks of age for 0.2%. Based on the small probability of having dose 1 before 6 weeks of age, we relaxed the requirement that infants be enrolled at birth to include infants enrolled by 6 weeks of age. We also dropped the requirement for a minimum duration of follow-up. This expanded group included 233,143 (85.7%) infants enrolled by 6 weeks of age (Figure 1, Cohort B). Cohort B was followed for 193,438 person-years. By including infants with incomplete follow-up, the proportion of vaccinated infants included in the analysis increased from 39.9% to 85.7%, and the proportion of infants with missing information for dose 3 was reduced from 60.1% to 32.8% (Table 1).

**Table 1:** **RotaTeq® (RV5) vaccine completion rates by cohort enrollment requirements and calendar year of first vaccination**

	**Overall**		**2006-2008**		**2009-2012**	
	**Cohort A** ^**1**^	**Cohort B** ^**2**^	**Cohort A**	**Cohort B**	**Cohort A**	**Cohort B**
	**n = 108, 533**	**n = 233,143**	**n = 49,360**	**n = 95,018**	**n = 59,173**	**n = 138,125**
Excluded due to insufficient enrollment (%)	60.1	14.3	55.4	14.2	63.3	14.4
Number completing vaccine series (three doses)	84,706	148,321	39,222	64,682	45,484	83,639
Vaccinated infants missing information for dose 3 (%)	60.1	30.8	55.4	26.9	63.3	33.4
Vaccine completion rates (%)	78.1	77.4	79.5	79.0	76.9	76.2
(95% CI)	(77.8-78.3)	(77.2-77.6)	(79.1-79.8)	(78.8-79.3)	(76.5-77.2)	(76.0-76.4)
Possible completion rates if infants with missing doses were all unvaccinated and all vaccinated (%)	31.1-91.2	54.5 - 85.3	35.4 - 90.8	58.4 - 85.3	28.2 - 91.5	51.8 - 85.2

^1^Cohort A: Enrolled at birth through first birthday.

^2^Cohort B: Enrolled by six weeks of age and no minimum follow-up.

The estimated completion rate among Cohort A infants with continuous enrollment from birth through the first year of life was 78.1% (95% CLs 77.8%, 78.3%; Table 1). Among Cohort B infants enrolled from 6 weeks of age, the estimated completion rate was 77.4% (95% CLs 77.2%, 77.6%; Table 1).

To assess the maximum potential impact of missing data due to incomplete follow-up, we estimated completion rates assuming that none and all of the infants with missing information on vaccinations due to incomplete follow-up received all 3 doses. For infants with continuous enrollment for the first year of life (Cohort A), the possible range of completion rates compatible with the data was 31.1% to 91.2% (Table 1). By including infants enrolled by 6 weeks of age and not requiring continuous enrollment for the first year of life (Cohort B), the possible range of completion rates decreased to 54.5% to 85.3% (Table 1).

Comparing the time periods from 2006–2008 to 2009–2012, for infants with complete follow-up we observed a decrease in completion rate from 79.5% (95% CLs 79.1%, 79.8%; Table 1) to 76.9% (95% CLs 76.5%, 77.2%; Table 1). For the unrestricted population allowing infants with incomplete follow-up, the completion rate declined from 79.0% (95% CLs 78.8%, 79.3%; Table 1) to 76.2% (95% CLs 76.0%, 76.4%; Table 1).

## Discussion

Automated claims databases offer researchers access to healthcare encounters for large populations followed over time. Two previous studies used automated claims databases to evaluate adherence to pentavalent rotavirus vaccination (RV5) by estimating the proportion of infants receiving at least 1 dose who went on to receive the complete schedule of 3 doses, and estimated completion rates of 79% and 83% [2,3]. These studies used a method known in clinical trials as a “complete case analysis” [7,8] which excludes from the analysis subjects with missing data due to incomplete follow-up who would otherwise meet the study inclusion criteria. Consequences of such exclusions include (a) a smaller study size and (b) potentially biased results if the outcomes (e.g., completion rates) of excluded subjects differ from subjects who are included [7,8]. In clinical trials, restricting analyses to subjects with complete data is widely discouraged owing to the potential for bias [5,7-9]. Instead, it is recommended that missing data should be quantified and that, at a minimum, sensitivity analyses should be performed to determine the potential impact of missing data on the results [7,9].

While restricting analyses to subjects with complete data is discouraged in clinical trials, complete case analysis is a common practice in automated database research. Further, the number of subjects excluded due to missing data often goes unreported, precluding any possibility of assessing the potential bias. The potential for bias would be a concern if a large number of subjects were excluded from analysis. If the proportion of subjects excluded is large, and their outcome rates differ from the outcome rates among those included, then study results would be biased. For instance, if a large proportion of infants were excluded and they were less likely to complete their vaccine schedule, the observed completion rates would be biased upward.

In light of these concerns, we used a claims database to address the potential impact of missing data due to incomplete follow-up on estimated completion rates of RV5 vaccination. After identifying the total number of vaccinated infants in the database, we determined that only 40% of infants receiving at least 1 dose of RV5 during the first year of life were continuously enrolled from birth through their first birthday. After including infants who received the first dose but who were not continuously enrolled during the first year of life, we were able to increase the proportion of vaccinated infants included in the analyses from 40% to 86%. Although the size of the population analyzed increased dramatically, the observed RV5 completion rates declined only slightly, from 78.1% to 77.4%. Completion rates varied little over time, suggesting temporal trends are not a major source of variation in completion rates during the study period. These results indicate that the impact of excluding infants with incomplete follow-up in the database was negligible when assessing RV5 completion rates in the insured population. Duration of follow-up in the claims database, therefore, appears to not be strongly related to the likelihood of receiving RV5 vaccination. If study populations across different claims databases used in previous studies were similar, excluding infants with incomplete follow-up in previous studies of RV5 would have had little effect on observed RV5 completion rates. By including in the analysis infants with incomplete follow-up, the range of possible completion rates was reduced by about 50%; nonetheless, the data are compatible with completion rates from 54.5-85.3%, indicating considerable uncertainty about completion rates among these infants after they disenroll from the database. This source of uncertainty due to missing data is not reflected in the conventional confidence limits, which capture only uncertainty due to random variation, and is an order of magnitude larger than random variation. Additional data on vaccination rates among uninsured infants are thus needed to understand the relation between insurance status and vaccination rates.

## Conclusion

In conclusion, restricting database analyses to infants with complete follow-up appears to give valid estimates of RV5 completion rates for this commercially insured population. Caution is warranted in generalizing beyond the insured population. Nonetheless, it is preferable to include in analyses subjects with incomplete follow-up [8]. In general, the assumption that a highly restricted population with complete follow-up will be representative of the entire insured population may not hold, hence we recommend adoption of more robust approaches to defining and analyzing study populations that account for missing data.

## Abbreviations

ACIP: Advisory committee on immunization practices
CL: Confidence limits
CPT: Current procedural terminology
HIRD: Healthcore integrated research database^SM^
RV1: Rotarix® monovalent human rotavirus vaccine
RV5: RotaTeq® pentavalent human rotavirus vaccine
